# Seroprevalence and Risk Factors for Hepatitis B Virus Infection in Adolescent Blood Donors within Selected Counties of Western Kenya

**DOI:** 10.1155/2020/8578172

**Published:** 2020-06-17

**Authors:** Hilary O. Awili, George C. Gitao, Gerald M. Muchemi

**Affiliations:** ^1^Department of Veterinary Pathology, Microbiology and Parasitology, University of Nairobi, P.O. Box 29053, Kangemi, Kenya; ^2^Department of Public Health, Pharmacology and Toxicology, University of Nairobi, P.O. Box 29053, Kangemi, Kenya

## Abstract

Hepatitis B virus is a widespread public health menace approximated to have infected 257 million people chronically by 2015. Data on the prevalence of HBV is important in formulating public health policies on HBV control like safe blood transfusion. Adolescents aged 15 to 24 years, known to engage in risky activities associated with HBV spread, constitute major blood donors in Kenya. Notwithstanding current blood donation safety measures, HBV still remain hazardous transfusion-transmissible infections in donated blood. This study therefore was to determine the prevalence of HBsAg and related risk factors among this donor group. A cross-sectional study was conducted from April 2019 to August 2019 in Siaya, Kisumu, and Homa Bay counties. One thousand (1000) voluntary blood donors 18 to 25 years old were recruited. A predonation questionnaire was used to record their sociodemographic features and prior risk exposures. Blood samples were initially tested for HBsAg using Murex HBsAg Version 3 (DiaSorin, UK) and positives confirmed using ARCHITECT HBsAg Qualitative Confirmatory assay (Abbott Ireland) as per the manufacturer's instructions. A result was considered positive if the first and confirmatory tests were all reactive. Generally, the prevalence of HBV was 3.4%, with no significant association between various sociodemographic variables and HBsAg positivity. Nevertheless, scarification and risky sexual behavior were significantly linked to HBV infections (odds ratio (OR) = 8.533, 95%confidence interval (CI) = 3.128‐23.275, *p* value of 0.001 and OR = 5.471, 95%CI = 1.925‐15.547, *p* value of 0.002, respectively). This study revealed a prevalence of 3.4% HBsAg among adolescent blood donors, with perilous sexual behaviors being the most significant risk factor, evidence that sexual contact still plays a major role in transmission of HBV among this donor group despite blood transfusion safety measures put in place. These study findings should therefore be put into consideration while framing health policies to mitigate effects of HBV infection on safe blood transfusion.

## 1. Introduction

Hepatitis B viral infection is a universal public health problem [1] approximated to have infected at least two billion people, with 257 million chronically infected by 2015 [2] and described as HBsAg-positive [3]. A developing African region with about 100 million infections bears the second major global problem of HBV chronic carrier rates after Asia [4]. Countries in this region, including Kenya, have intermediary chronic HBV populace infection rates of 2%-7% or greater at ≥8% [1]. Although a gradual general decline in HBsAg infection rates in most countries has been reported, there is a remarkable increase in some African and Western countries [3]. However, the true rate of infection in these countries cannot be ascertained because of the intermittent and poor recording of incidence. Besides, very few studies have been done to elucidate the occurrences in the sub-Saharan African region, despite the importance of knowledge of HBsAg prevalence in the organization as well as making public health choices regarding hepatitis B control measures such as safe blood transfusion and vaccination. Transfusion-transmissible infections such as HBV remain hazardous in donated blood in spite of the current blood donation safety developments put in place [2]. This situation is more endemic in African countries such as Kenya [5] where it is further compounded by the fact that donor blood is majorly sourced from adolescents aged 15 to 24 years in learning institutions [6]. This period in life is known to mark the onset of very active and risky sexual behaviors and experiment with intravenous drug use, tattoos, and other body piercing tools, all increasing risks of exposure to HBV infection [7], making the adolescent stage a period of high vulnerability to the disease. There is a need for sustained research and documentation of HBV prevalence especially among major donor groups like the youth to ensure donor blood safety. While the majority of studies conducted on the prevalence of HBV have focused on HIV patients, blood donors in general, and the general population, very few have focused on adolescents in Kenya. This study consequently sought to document the occurrence of HBsAg as well as associated risk factors in youths aged 18 to 25 years in Kisumu, Siaya, and Homa Bay counties within Western Kenya.

## 2. Materials and Methods

### 2.1. Study Design, Setting, and Population

**Figure 1:**
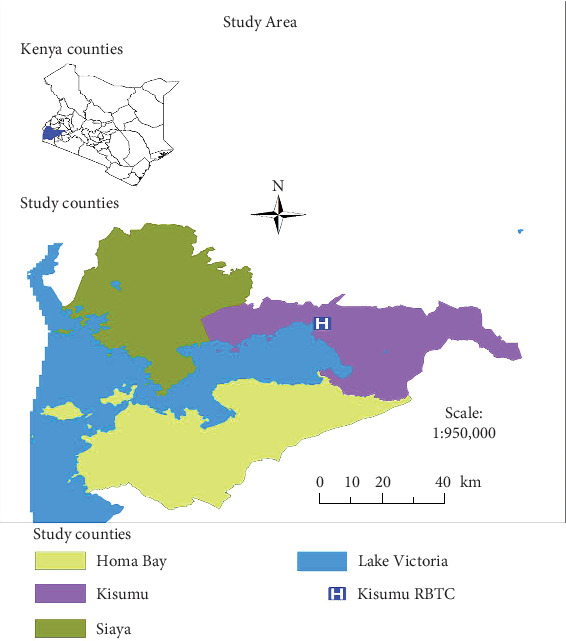
Map showing the study areas of Kisumu, Homa Bay, and Siaya counties and the regional blood transfusion center (RBTC).

A cross-sectional study was conducted from April 2019 to August 2019 within three counties of Kisumu, Siaya, and Homa Bay in Western Kenya (Figure 1). These counties serve as the major donor blood source for most hospitals in the Western region. Besides, these counties have the highest HIV infection rates and have recorded perilous sexual behavior among adolescents, intravenous drug practices, and other activities that have been related to high levels of transmission and infection with HBV [6, 8]. It is on this basis that these areas were selected for the study. Laboratory processes were performed in the Virology Laboratory at the Kisumu Regional Blood Transfusion Center (RBTC). Using Fisher's formula and based on KNBTS selection protocol, 1000 consenting adolescent voluntary blood donors between 18 and 25 years old from different learning institutions were recruited for the study. A standard National Blood Transfusion Service questionnaire was administered in English or Kiswahili. Interviews were conducted using the questionnaire with each study subject questioned on sociodemographic characteristics and exposure to various risk factors. For ethical reasons and integrity of the test process, a bar-coded donor identity was then used to link each donor questionnaire to the blood samples collected.

### 2.2. Blood Collection and Serological Analysis

After donor blood collection, whole blood samples from donor bags were dispensed into red top vacutainer tubes and left to clot at room temperature. Thereafter, 75 *μ*l of serum was pipetted from the vacutainer tube for analysis using Murex HBsAg Version 3 (manufactured by DiaSorin S.p.A. UK Branch) as per the manufacturer's instructions. Samples that turned positive for HBsAg using this initial test were shipped to the Regional Blood Transfusion Laboratory in Nairobi, where a confirmatory test was done by Chemiluminescence Immunoassay (CLIA) using the ARCHITECT HBsAg Qualitative Confirmatory assay (Abbott Ireland, Diagnostics Division, Sligo, Ireland) (1P98) as per the manufacturer's instructions. A result was considered positive if the first test and the confirmatory test were all reactive.

### 2.3. Data Management and Statistical Analysis

The data generated was entered into MS Excel, cleaned, and analyzed using SPSS software version 24. Presentation of descriptive statistics was done in frequencies and percentages by the use of tables and charts. A Chi-squared test was used to determine the statistical significance between the risk factors and the outcome variable with a *p* value ≤ 0.05 considered statistically significant. The association between HBsAg seroprevalence and various risk factors was established using logistic regression where odds ratios (OR) with their 95% confidence intervals (CI) aided to examine the effect of the various risk factors on the occurrence of HBsAg seropositivity, with a *p* value < 0.05 regarded as significant.

### 2.4. Ethics

The study obtained approvals from the Kenya National Blood Transfusion Management and the University of Nairobi Board of Postgraduate Studies. Confidentiality of the study participants was safeguarded through the use of codes and ignoring materials that pinpointed at the study subjects. Participants who were found positive for HBsAg were confidentially informed of their status and referred to local hospitals for treatment.

## 3. Results

### 3.1. Seroprevalence of HBsAg and Sociodemographic Characteristics of Study Participants

A total of 1000 adolescent voluntary blood donors aged between 18 years and 25 years, spread across the counties Kisumu, Siaya, and Homa Bay, were enlisted into the study. Of the thirty-seven samples that initially tested positive for HBsAg, thirty-four were confirmed HBsAg-positive, giving an overall seroprevalence of 3.4%. The two test methodologies used as initial and confirmatory tests in this study were notably applied because of the good analytical agreement between the methods and could therefore reliably detect and confirm HBV infection in the study population. Nevertheless, some discrepancies in quantitative measurement as reported between the initial test (*N* = 37) and the confirmatory test (*n* = 34) may have been due to factors such as variations in the standard calibrators used in each assay, difficulties in quality assurance occasioned by sample transport, assay degeneration, and operator error. Comparatively across the counties, seroprevalence was highest in Siaya at 1.3% (*n* = 13) followed by Homa Bay at 1.1% (*n* = 11) while Kisumu recorded the least at 1.0% (*n* = 10). Generally, there was no statistically significant association between sociodemographics such as age, age group, gender, county of origin, and number of donations to HBsAg test results. This is summarized in Table 1.

### 3.2. Risk Factors Associated with HBsAg in Adolescent Blood Donors

The risk factors associated with HBsAg seroprevalence in adolescent blood donors in this study are summarized in Table 2. Thirty-one (3.1%) adolescent blood donors had a history of high-risk sex behavior such as having multiple sex partners, sex without use of condoms, and having sexual intercourses in exchange for cash. Six (19.4%) out of this number were positive for HBsAg. On bivariate logistic regression analysis, hepatitis B virus infection and high-risk sex behavior showed a statistically significant association (OR = 8.066, 95%CI = 3.067‐21.214, *p* = 0.001).

**Figure 2:**
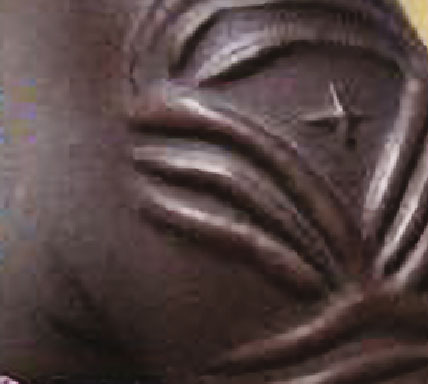
Tattooing marks shown by one of the adolescent blood donors in Homa Bay county.

**Figure 3:**
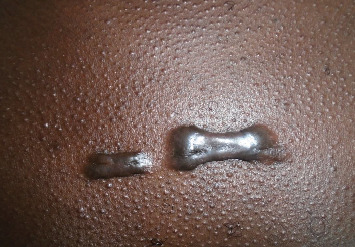
Scarification marks resulting from an incision of the skin made by a traditional healer as shown by one adolescent donor in Siaya county.

Scarification was reported among 38 (3.8%) adolescent blood donors, some with visible scarification marks on their bodies as a result of traditional practices that involved making cuts on their bodies to deliver traditional medicines, while some had tattoo marks made on them for aesthetic appeal (Figures 2 and 3). A total of five (13.2%) of this group were confirmed HBsAg-positive cases. Bivariate logistic regression found a statistically significant relationship between HBV disease and scarification (OR = 4.875, 95%CI = 1.774‐13.392, *p* = 0.002).

Twenty-one (2.1%) of the study participants reported a history of past accidental injuries such as needle sticks, knife cuts, or accidental cuts acquired from barbershops. Only 3 (14.3%) of this group tested positive for HBsAg, giving a statistically significant link between HBV disease state and past injuries (OR = 5.097, 95%CI = 1.426‐18.213, *p* = 0.012) on bivariate analysis. Intravenous drug use was reported in 15 (1.5%) donors among which only one was a confirmed HBsAg-positive, giving no statistically important association linking intravenous drug use to HBV infection (OR = 2.061, 95%CI = 0.263‐16.139, *p* = 0.491). In multivariate analysis of a selection of variables for independent determinants of HBsAg positivity in adolescent blood donors, only past risky sexual activities (OR = 8.533, 95%CI = 3.128 to 23.275) and scarification (OR = 5.471, 95%CI = 1.925 to 15.547) remained statistically significant predictors of HBsAg positivity (Table 2) with *p* values of 0.001 and 0.002, respectively.

## 4. Discussions

This research was set to determine the seroprevalence and risk factors associated with HBsAg infection among adolescent blood donors between 18 and 25 years old in Kisumu, Siaya, and Homa Bay counties in Western Kenya. The overall seroprevalence among adolescent blood donors in the three counties was 3.4%. The HBsAg seroprevalence of 3.4% reported in this study is suggestive of intermediary endemicity and increased predisposition to HBV infections among adolescent blood donors. This prevalence rate falls within the range of hepatitis B prevalence in sub-Saharan Africa viewed as intermediate to high endemic areas with ≥2% to ≥5% rates of infection [9, 10]. The finding is comparably lower than a 3.9% seroprevalence earlier reported in a study across the general population in the same area [11] but slightly higher than a 3.1% seroprevalence reported in a study to determine the incidences and determinants of HBV virus disease among teenagers in Enugu, Nigeria [12]. Furthermore, the 3.4% seroprevalence of HBsAg among adolescent blood donors reported in the three counties in the current study should be worrying since HBsAg appears to persist among the adolescent donor population despite the blood donation safety enhancements reportedly laid out across the regional blood transfusion centers in the country [13]. This is an indicator that the efforts so far put to improve donated blood safety are still not adequate in ensuring donor blood is safe for transfusion.

In the analysis of risk factors, high-risk behavior and scarification were significantly associated with HBV infection while a history of drug use and injuries such as accidental needle sticks was reported but remained insignificantly associated with HBV infection. Donors who stated having been involved in high-risk sex activities such as having multiple sex partners, sexual intercourses without condom use, and sex in exchange for money were almost eight times more likely to test positive for HBsAg than those with no history of high-risk sex behavior, making high-risk sex behavior the most significant predictor of HBsAg positivity in the study. The findings here corroborate a previous local study on the seroprevalence and determinants of TTIs among voluntary blood donors in Kisumu, Siaya, and Homa Bay counties [14] which reported a significant association between high-risk sex behavior and HBV infection. Likewise, this study finding is comparable to studies in other regions such as Nigeria and Egypt [15–17] that have also associated history of risky sexual behaviors such as sexual intercourses with no condom use and having sexual intercourses in exchange for money in the dynamics of the spread of HBV.

Scarification is another putative risk factor that had a meaningful link to HBsAg positivity in the current study. Although indisputable studies absolutely attributing the acquisition of HBV to scarification are hardly available, this study reported a significant association between HBsAg positivity and scarification, with 5 (13.2%) of the 38 study subjects with scarification marks testing positive for HBsAg. Some of the donors in this current study who had visible scarification marks on their bodies on further probing reported that the marks were a result of traditional practices that involved making a cut on the skin to deliver herbal medicines for relief from distinct medical conditions, while some had modern body tattoos for aesthetic appeal. These traditional scarifications mainly done at the community level by traditional doctors involve repeated use of unsterile instruments which have the potential of triggering hemorrhage and skin ulcerations, intensifying the possibility of percutaneous spread of hepatitis B virus. Furthermore, in modern society, tattooing is done as a form of body modification for beauty and aesthetic appeal. This practice is very common among the adolescent and is achieved through processes that involve cut through the skin, removal of skin parts, chemical imprinting, and other techniques that may cause skin lacerations. This process involves the use of various equipment that requires standard sterilization procedures to prevent horizontal transmission of viral infections such as HBV. However, sterilization procedures are seldom followed. It is therefore plausible to suggest that the involvement of the adolescents in such practices as reported in this study could have exposed them to the horizontal transmission of HBV. The findings in this current study are corroborated with reports in other research findings where hepatitis B virus is implicated as one of the pathogens transmissible through use of infected tools during traditional practices involving skin scarification or tattooing in modern society [18–20]. Tattooing as well as other skin-penetrating practices has also remained characteristically associated with HBV disease in a number of studies [21–23]. In the current study, intravenous drug use and history of injuries such as needle sticks did not show any significant association with hepatitis B seropositivity among adolescent blood donors. Only one out of the 15 respondents who admitted having used intravenous drugs was a confirmed positive HBsAg case, with no significant link between HBsAg positivity and intravenous drug use. This very low number of respondents who admitted having used intravenous drugs could be attributed to the fact that not many participants were willing to divulge information about past use of injectable drugs, possibly due to stigma associated with intravenous drug use and criminalization of the use of these drugs. The number of reported intravenous drug users among participants in the current study could possibly go high if the barriers of stigma and criminalization are removed. Nevertheless, the finding in this current study compares similarly to a previous study in the same area [14] that reported no significant association between TTIs including HBV and previous exposure to illicit drug use. In the current study, history of injuries such as accidental needle sticks has recorded no significant association with HBV infection. Only 3 (14.1%) of the 21 (2.1%) study participants reported having had injuries due to various causes such as needle sticks and other percutaneous exposures such as accidental knife cuts or small cuts acquired from barbershops. In contrast, study findings in other areas [23, 24] have reported a significant link between history of accidental injuries, especially needle sticks, and HBV infection.

## 5. Conclusions

The study revealed a seroprevalence of 3.4% HBsAg among adolescent blood donors in Kisumu, Siaya, and Homa Bay counties. Risk factors identified to be independently associated with HBsAg positivity are high-risk sex behavior and scarification, with the most reported risk factor being high-risk sex behaviors. This is evidence that sexual contacts play a major role in the transmission of HBV among this group of blood donors. The study reports a significant HBsAg infection rate among a key donor population despite recent blood donation safety improvements put in place to ensure safe blood transfusion; therefore, this study finding should be taken into consideration while framing health policies geared towards mitigating the effect of HBV infection on safe blood transfusion. Fostering safe sex education in learning institutions to minimize high-risk sex behaviors, policy changes to incorporate early uptake of HBV testing, and subsequent vaccination of adolescents as a subgroup at high risks of HBV infection should be considered to help minimize the burden of HBV infection observed among this blood donor group. In addition, employment of high-quality screening assays combined with strict predonation screening of blood donors including adolescents should be continuously implemented to improve blood safety and availability.

## Figures and Tables

**Table 1:** HBsAg frequency in relation to sociodemography in adolescent blood donors in Kisumu, Homa Bay, and Siaya counties.

Characteristic	Nonreactive, *n* (%)	Reactive, *n* (%)	*p* value
Age brackets			0.584
18-21	851 (85.1%)	31 (3.1%)	
22-25	115 (11.5%)	3 (0.3%)	
Gender			0.470
Male	599 (59.9%)	19 (1.9%)	
Female	367 (36.7%)	15 (1.5%)	
Number of donations			0.158
First	928 (92.8%)	31 (3.1%)	
Repeats	38 (3.8%)	3 (0.3%)	
Counties of origin			0.458
Kisumu	371 (37.1%)	10 (1.0%)	
Homa Bay	311 (31.1%)	11 (1.1%)	
Siaya	284 (28.4%)	13 (1.3%)	

**Table 2:** Risk factors associated with HBsAg among adolescent blood donors in Kisumu, Homa Bay, and Siaya counties.

Risk factor	Total donors (*N* = 1000)	HBsAg (*N* = 34) ^∗^Positive (%)	Bivariate OR (95% CI)	Multivariate OR (95% CI)	*p* value
High-risk sex behavior	31 (3.1%)	6 (19.4%)	8.066 (3.067-21.214)	8.533 (3.128-23.275)	0.001
Scarification	38 (3.8%)	5 (13.2%)	4.875 (1.774-13.392)	5.471 (1.925-15.547)	0.002
History of injury	21 (2.1%)	3 (14.3%)	5.097 (1.46-18.213)	2.912 (0.736-11.523)	0.128
Intravenous drug use	15 (1.5%)	1 (6.7%)	2.061 (0.263-16.139)	1.256 (0.147-10.750)	0.835

## Data Availability

Any additional data is available upon request through the corresponding author, Hilary Awili, through awilihilary@students.uonbi.ac.ke and daniowello@gmail.com.

## Additional Points

The data available for this study to evaluate the infectious risks posed by occult HBV infections in this study population was limited by the unavailability of some test methods. The study was dependent on two test methods (ELISA and CLIA) to test and confirm the presence of disease but could not employ molecular amplification techniques to detect HBV DNA to identify occult HBV infections within the study population.
